# Exosome-based *Ldlr* gene therapy for familial hypercholesterolemia in a mouse model

**DOI:** 10.7150/thno.49874

**Published:** 2021-01-01

**Authors:** Zhelong Li, Ping Zhao, Yajun Zhang, Jia Wang, Chen Wang, Yunnan Liu, Guodong Yang, Lijun Yuan

**Affiliations:** 1 Department of Ultrasound Diagnostics, Tangdu Hospital, Fourth Military Medical University, Xi'an, People's Republic of China; 2 The State Laboratory of Cancer Biology, Department of Biochemistry and Molecular Biology, Fourth Military Medical University, Xi'an, People's Republic of China; 3 Department of Ultrasound Diagnostics, Beijing Tongren Hospital, Capital Medical University, Beijing 100730, China

**Keywords:** familial hypercholesterolemia, atherosclerosis, exosomes, low-density lipoprotein receptor, gene therapy

## Abstract

Familial hypercholesterolemia (FH), with high LDL (low-density lipoprotein) cholesterol levels, is due to inherited mutations in genes, such as low-density lipoprotein receptor (LDLR). Development of therapeutic strategies for FH, which causes atherosclerosis and cardiovascular disease, is urgently needed.

**Methods:** Mice with low-density lipoprotein receptor* (Ldlr)* deletion (*Ldlr*^-/-^ mice) were used as an FH model. *Ldlr* mRNA was encapsulated into exosomes by forced expression of *Ldlr* in the donor AML12 (alpha mouse liver) cells, and the resultant exosomes were denoted as Exo^Ldlr^. *In vivo* distribution of exosomes was analyzed by fluorescence labeling and imaging. The delivery efficiency of *Ldlr* mRNA was analyzed by qPCR and Western blotting. Therapeutic effects of Exo^Ldlr^ were examined in *Ldlr*^-/-^ mice by blood lipids and Oil Red O staining.

**Results:** The encapsulated mRNA was stable and could be translated into functional protein in the recipient cells. Following tail vein injection, exosomes were mainly delivered into the liver, producing abundant LDLR protein, resembling the endogenous expression profile in the wild-type mouse. Compared with control exosomes, Exo^Ldlr^ treatment significantly decreased lipid deposition in the liver and lowered the serum LDL-cholesterol level. Significantly, the number and size of atherosclerotic plaques and inflammation were reduced in the Exo^Ldlr^-treated mice.

**Conclusions:** We have shown that exosome-mediated *Ldlr* mRNA delivery effectively restored receptor expression, treating the disorders in the *Ldlr*^-/-^ mouse. Our study provided a new therapeutic approach for the treatment of FH patients and managing atherosclerosis.

## Introduction

Familial hypercholesterolemia (FH) is an autosomal dominant genetic disease characterized by severely elevated plasma low-density lipoprotein (LDL) cholesterol (LDL-C) and premature coronary heart disease 1. Most FH patients (about 95% of cases) carry a functional loss mutation of the LDL receptor (LDLR) gene 2. The prevalence of the heterozygous mutations in LDLR has been estimated at 1 in 200 to 500 in the population, and the homozygous form at 1 in 100, 000 individuals 3. As a key lipoprotein receptor on the surface of hepatocytes, the LDLR is critical for LDL-C clearance from the circulation by the liver. By endocytosis and further processing of the LDL-C, LDLR removes most excess LDL-C from the serum 4, and there are no other proteins have similar functions as LDLR *in vivo* 5,6. The heterozygotes (HeFH) typically have twice the normal plasma LDL levels and cardiovascular diseases at an early age 7. Homozygous individuals (HoFH) face much higher LDL-C levels and often die before the age of 20 years if untreated 8. Although existing therapeutics, such as statins, ezetimibe, and PCSK9 inhibitors, have some beneficial effects in HeFH 9, few drugs are effective in HoFH even at high-doses 10. Lipid apheresis and liver transplantation 11 are the current clinical managements to reduce the LDL-C level, while gene therapy holds promise.

Accumulating evidence supports the idea that the overexpression of LDLR in the liver by retrovirus 12, adenovirus 13, or AAV 14 could reduce total cholesterol efficiently. A recombinant LDLR-expressing AAV8 vector is currently in phase II clinical trials (ClinicalTrials.gov NCT02651675). However, intrinsic carcinogenicity, cytotoxicity, and immunogenicity limit its clinical application 15.

Exosomes are small intracellular vesicles ranging from 30-150 nm in size and have an important role in cell-cell communication 16. Many studies have shown that exosomes can efficiently deliver cargos, such as mRNA, miRNA, and even plasmid DNA, to target cells, and are emerging as a promising therapeutic carrier 17-19. Compared with viruses, exosomes, as “natural nanoparticles”, are easy to handle, non-cytotoxic, and non-immunogenic 20. It is thus promising to develop an exosome-based LDLR-gene delivery strategy and explore the therapeutic effects on HoFH.

In this study, exosomes encapsulating abundant *Ldlr* mRNA were engineered by forced expression of *Ldlr* in the donor liver cells. The encapsulated *Ldlr* mRNA was stable and functional in the recipient cells. Using the *Ldlr*^-/-^ mouse model, we found that exosomes-mediated *Ldlr* mRNA delivery could robustly restore LDLR expression and thus reverse the phenotype, such as steatosis, high LDL cholesterol, and atherosclerosis. The study has offered a new therapeutic approach as an alternative to the viral vectors for the treatment of FH patients. Moreover, it also shed light on the management of atherosclerosis and other genetic diseases associated with liver abnormity.

## Results

### Construction and characterization of Exo^Ldlr^

**Figure 1:**
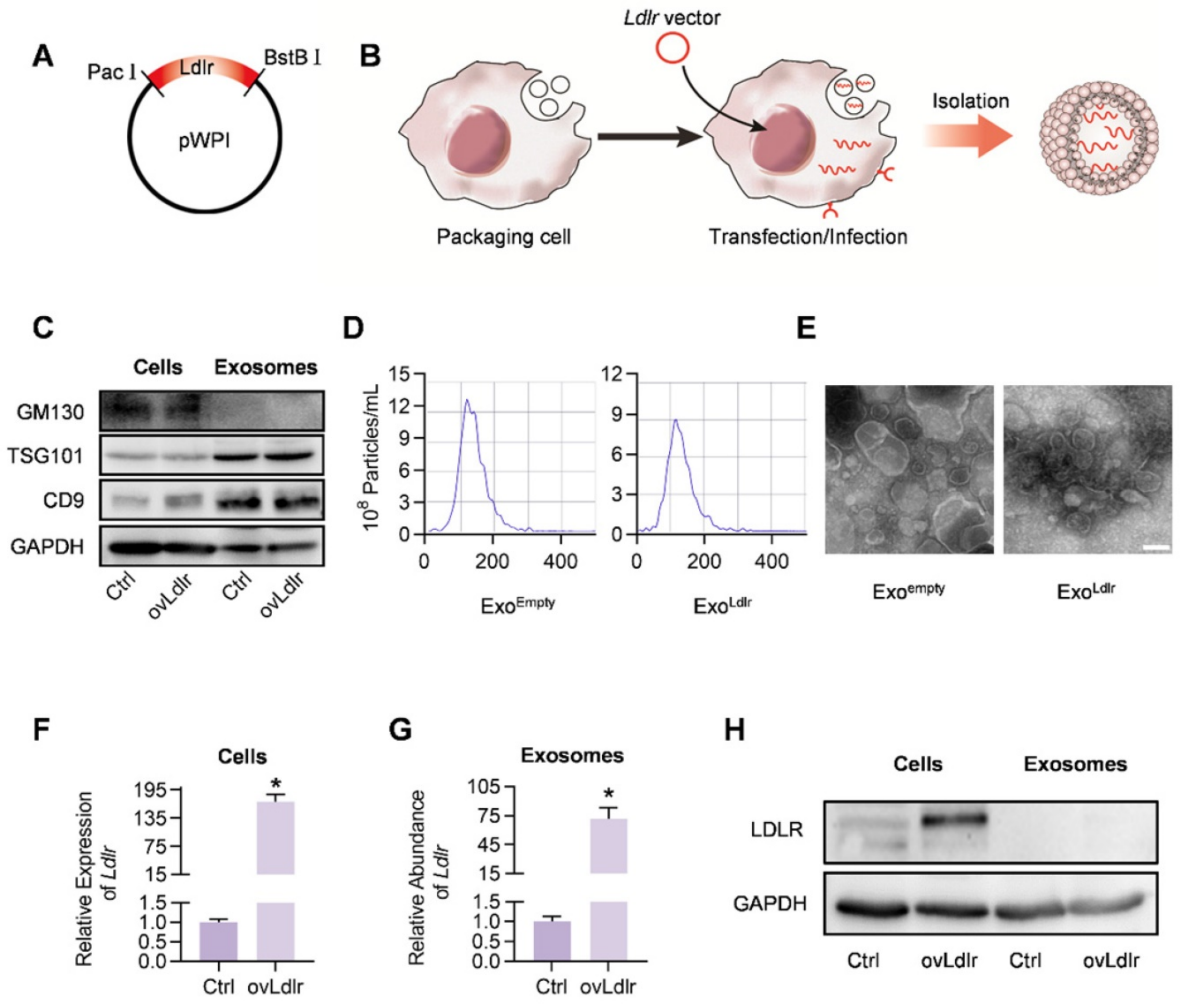
** Construction and characterization of Exo^Ldlr^.** A. Cloning of *Ldlr*-expressing plasmid. The CDS of *Ldlr* was cloned into the plasmid backbone with restriction endonuclease sites as indicated. B. Schematic illustration of *Ldlr* mRNA encapsulation procedure into the exosomes. The donor cells were forced to express *Ldlr* upon transfection of the plasmid or infection with the *Ldlr*-expressing virus. The high level of *Ldlr* was thus passively enriched in the exosomes. C. Western blot analysis of the exclusive and inclusive exosome markers in the isolated exosomes and parental cells. Cells were transfected/infected with control or *Ldlr* vector. D. Size distribution of the indicated exosomes analyzed by ZetaView Particle Metrix. E. Representative transmission electron microscope images of the indicated exosomes. Scale bar=100 nm. F. Expression of *Ldlr* mRNA in AML12 cells treated as indicated. GAPDH served as an internal control. Data are expressed as mean ±SEM of three independent experiments. *, p< 0.05 by t-test. G. *Ldlr* mRNA abundance in exosomes derived from AML12 cells treated as indicated. H. Western blot analysis of LDLR at the protein level in parental cells and derived exosomes. Cells were transfected with control or *Ldlr* vector. GAPDH served as the loading control. Representative data of 3 independent experiments.

A robust abundance of the targeted cargo is a prerequisite for exosome-mediated gene therapy. To generate *Ldlr* mRNA-enriched exosomes, an *Ldlr*-expressing vector was constructed, in which the *Ldlr* coding sequence (CDS) was cloned downstream of the EF1α promoter and fused to the Woodchuck Hepatitis Virus (WHP) Post-transcriptional Regulatory Element (WPRE), creating a tertiary structure with enhanced expression when transcribed (Figure 1A). Upon transfection or infection of the vectors into the packaging AML12 cells, the transcribed *Ldlr* mRNA would be passively loaded into the exosomes (Figure 1B). Western blot analysis revealed a similar expression pattern of the inclusive and exclusive exosomal markers in cells and derived exosomes between control and *Ldlr* over-expressing groups (Figure 1C). Moreover, nanoparticle tracking analysis (Figure 1D) and transmission electron microscopy (Figure 1E) showed that Exo^empty^ and Exo^Ldlr^ were similar in size distribution and morphology, ranging from 30-150 nm in diameter.

Next, we examined whether the *Ldlr* mRNA and protein were efficiently encapsulated into the exosomes. As expected, *Ldlr* mRNA was increased more than 100-fold upon *Ldlr* overexpression (Figure 1F). A similar increase in the *Ldlr* mRNA copies was observed in the derived exosomes (Exo^Ldlr^) (Figure 1G). However, LDLR protein was rarely seen in either the control Exo^empty^ or Exo^Ldlr^, though abundant LDLR protein was found in the donor cells (Figure 1H). The *Ldlr* mRNA encapsulated in the exosomes remained stable after long time preservation (Figure S1), probably because the membrane structure provided protection from the RNase. These data indicated that the *Ldlr* mRNA, rather than the protein, was successfully and efficiently loaded into the exosomes by its forced expression in donor cells.

### Exo^Ldlr^ efficiently delivers functional *Ldlr* mRNA into recipient cells

**Figure 2:**
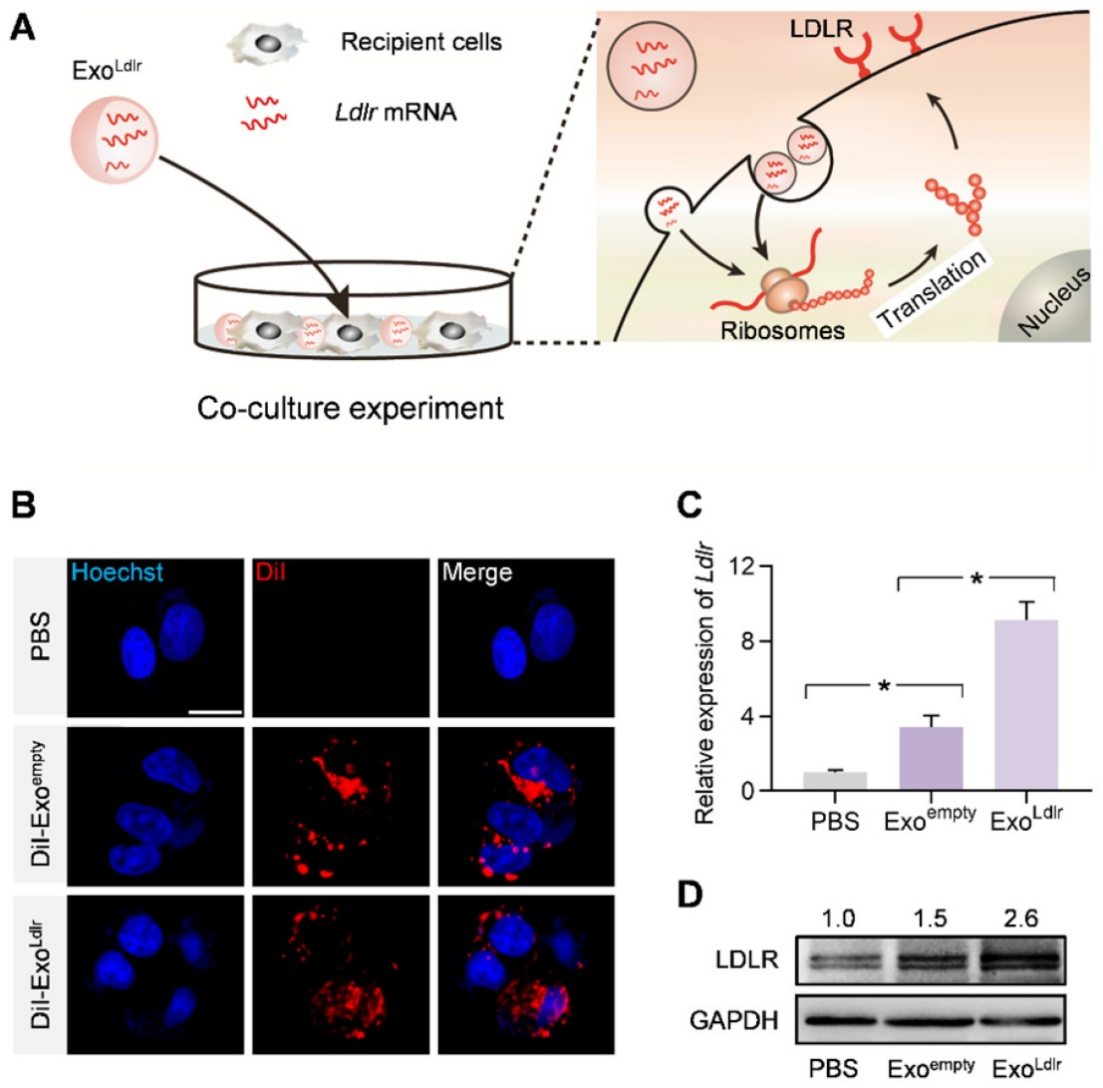
** Exo^Ldlr^ efficiently delivers functional *Ldlr* mRNA *in vitro*.** A. Schematic illustration of the exosome-mediated *Ldlr* mRNA delivery into the recipient cells, where the mRNA is translated into the functional protein. B. Fluorescence microscopy images showing the endocytosis of exosomes in the recipient cells. The intracellular distribution of DiI-labeled exosomes was analyzed by fluorescence microscopy. Nuclei were counterstained with Hoechst. PBS served as the negative control. Scale bar=5 μm. C. qPCR analysis of *Ldlr* mRNA expression in HEK 293T cells treated as indicated. Data are expressed as mean ± SEM of three independent experiments. *, p< 0.05 by one-way ANOVA. D. Western blot analysis of LDLR protein expression in HEK 293T cells treated as indicated. GAPDH served as the loading control. Representative data from three independent experiments.

We investigated whether *Ldlr* mRNA encapsulated in the exosomes could be efficiently delivered and translated into the functional protein in recipient cells. HEK 293T cells with low endogenous *Ldlr* expression were incubated with DiI-labeled Exo^empty^ or Exo^Ldlr^ (Figure 2A). Fluorescence microscopy revealed efficient endocytosis of both exosomes by HEK293T cells to a similar extent (Figure 2B). *Ldlr* mRNA level was significantly increased in cells receiving Exo^Ldlr^ treatment (Figure 2C). Notably, there was also a slight increase in *Ldlr* mRNA in the Exo^empty^ group, probably because the exosome donor AML12 cells express high levels of *Ldlr*. Consistently, the LDLR protein was significantly enhanced upon Exo^Ldlr^ treatment (Figure 2D). Moreover, the *Ldlr* mRNA delivered into recipient cells peaked at about 24 h and decreased gradually with time (Figure S2). To further confirm the functional effects of exosome-mediated *Ldlr* mRNA delivery on lipid uptake, cells treated with the indicated exosomes were further incubated with DiI-labeled LDL in AML12 hepatocytes. As shown in Figure S3, Exo^Ldlr^ treatment significantly promoted lipid (LDL) uptake and thus clearance from the medium. These data indicated that Exo^Ldlr^ effectively delivered functional *Ldlr* mRNA into recipient cells.

### Exo^Ldlr^ treatment restores LDLR protein expression *in vivo*

**Figure 3:**
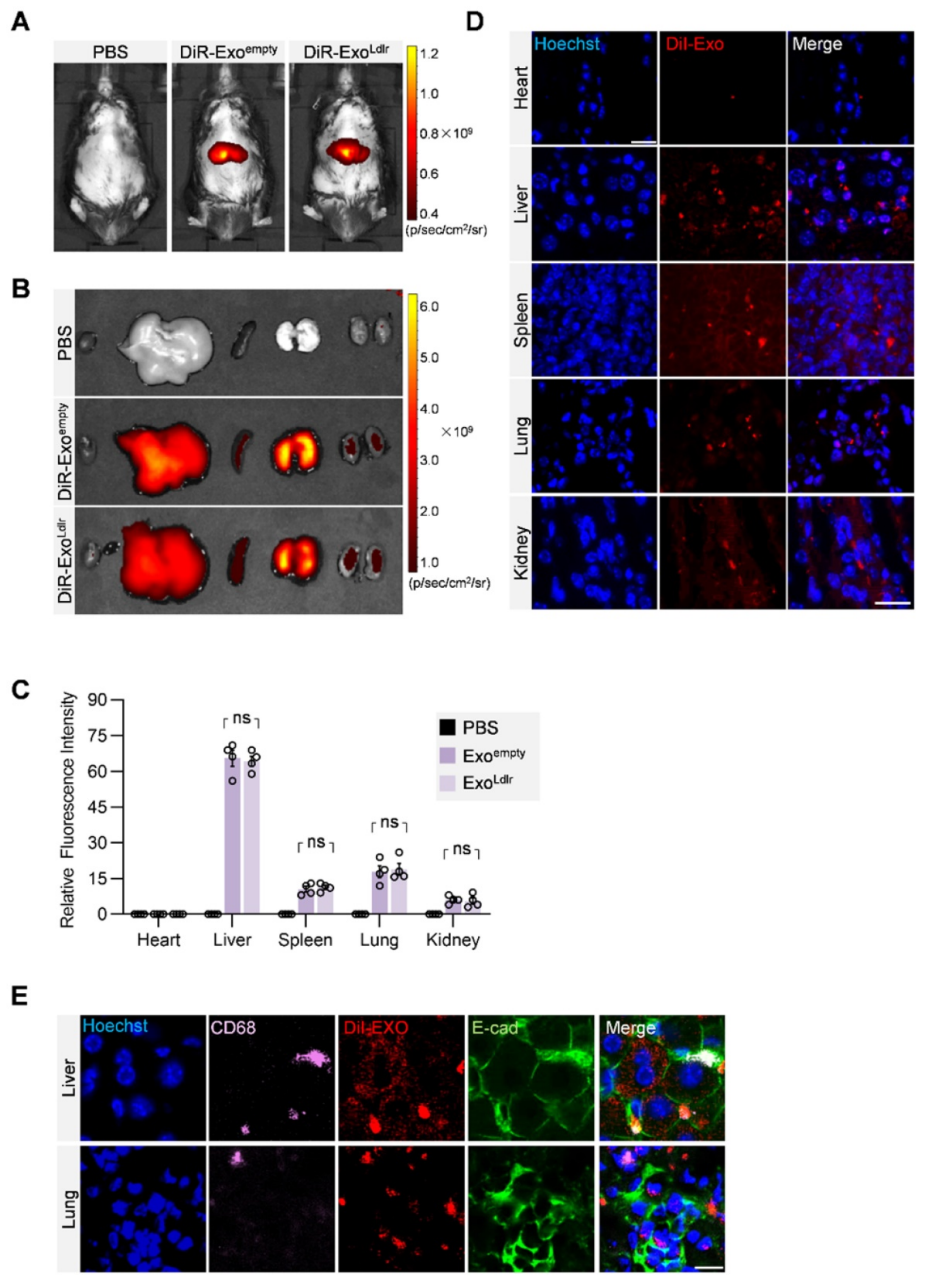
** In vivo distribution of Exo^Ldlr^ after tail vein injection.** A. Representative IVIS images of mice injected with 100 μL PBS, 100 μg (in 100 μL) DiR labeled Exo^empty^ or Exo^Ldlr^ via the tail vein. IVIS imaging was performed 4 h after injection. B. *Ex vivo* fluorescence imaging analysis of the distribution of the DiR-labeled exosomes in different organs, including the liver, spleen, heart, kidney, and lung. C. Quantification of the fluorescence signal intensity in Fig 3B. n=4, ns, no significance. D. Representative fluorescence microscopic images of the localization of DiI-labeled exosomes. Mice were injected with 100 μL DiI-labeled Exo^empty^ or Exo^Ldlr^ via tail vein and sacrificed 4 h after injection. Scale bar = 20 μm. E. Representative images of localization of the exosomes, immune cells, and the epithelial cells in the liver or lung sections. Scale bar = 10 μm.

To profile the distribution of the exosomes *in vivo*, DiR-labeled exosomes were tracked. In vivo imaging system (IVIS) demonstrated that both DiR-labeled Exo^empty^ and Exo^Ldlr^ were mainly delivered into the liver, spleen, and lung (Figure 3A-C). Fluorescence microscopy analysis of the DiI-labeled exosomes in the tissue sections further confirmed that the encapsulation of *Ldlr* mRNA did not affect the *in vivo* distribution profiles of Exo^empty^ and Exo^Ldlr^ (Figure 3D). Moreover, the exosomes were taken up by both epithelial (co-localization of the exosomes with the epithelium marker E-cadherin) and immune cells (co-localization of the exosomes with the macrophage marker CD68) (Figure 3E).

**Figure 4:**
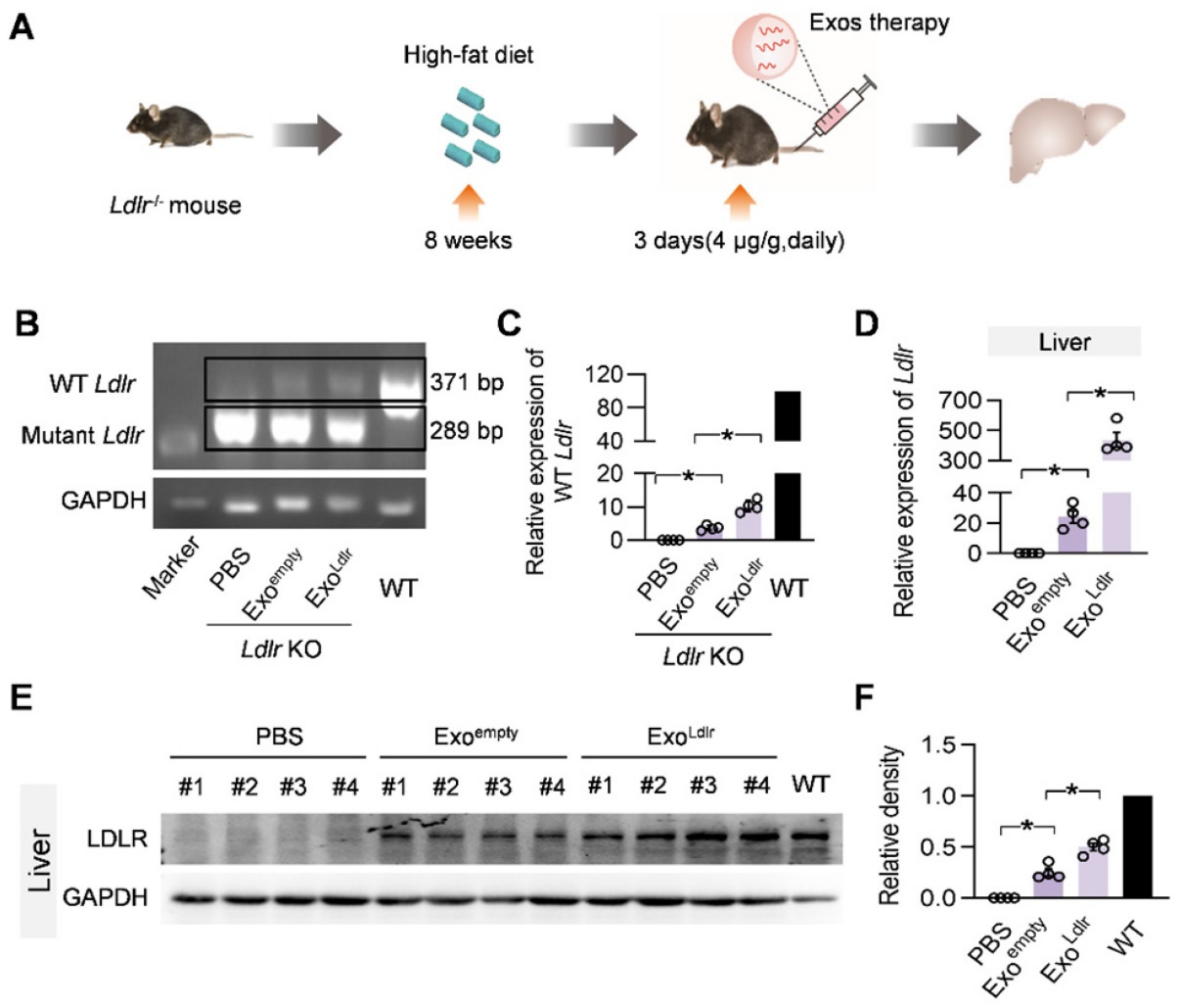
** Exo^Ldlr^ effectively delivers *Ldlr* mRNA into the liver in vivo.** A. Schematic illustration of the experimental procedure. *Ldlr*^-/-^ mice were fed with a high-fat diet for 8 weeks, followed by the injection of indicated exosomes. The expression of *Ldlr* at both mRNA and protein levels in the liver was examined 3 days after injection. B. Semi-quantitative PCR analysis of *Ldlr* mRNA expression in livers from mice treated as indicated. Lane 1 is DNA ladder. The lower 289 bp band represents the endogenous truncated *Ldlr* from knockout mice and the 371 bp band represents the exogenous wild-type *Ldlr*. Data shown are representative of 4 independent experiments. C. Quantitative data of Figure 4B. Data are expressed as mean ± SEM. *, p < 0.05 by one-way ANOVA. D. qPCR analysis of the wild-type *Ldlr* mRNA in livers from mice treated as indicated. n=4. Data are expressed as mean ± SEM. *, p < 0.05 by one-way ANOVA. E. Western blot analysis of the LDLR expression at the protein level in livers from mice treated as indicated. Notably, there was no endogenous LDLR protein expression in the *Ldlr*^-/-^ mice with PBS treatment. F. Quantification of Western blot bands by densitometry. Data are expressed as mean ± SEM. *, p < 0.05 by one-way ANOVA.

To further confirm whether Exo^Ldlr^ could deliver functional *Ldlr* mRNA *in vivo*, *Ldlr*^-/-^ mice fed with a high-fat diet for 8 weeks were injected with Exo^empty^ or Exo^Ldlr^ (Figure 4A). Semi-quantitative PCR analysis revealed an 82 bp larger, wild-type *Ldlr* band together with the endogenous truncated band (Figure 4B-C). To differentiate the wild-type *Ldlr* mRNA from the endogenous mutant *Ldlr*, a nested real-time PCR assay was developed to increase the sensitivity and specificity. The external primer set spanned the deleted region, while the internal primer set was localized within the deleted region (Figure S4A-B). qPCR data confirmed the successful delivery of wild-type *Ldlr* into the liver (Figure 4D). Consistent with the mRNA delivery, Western blot analysis revealed successful LDLR expression at the protein level in livers from mice treated with Exo^Ldlr^ (Figure 4E-F). Notably, Exo^empty^ delivery also produced significant LDLR expression in the liver, though at a much lower level than that of Exo^Ldlr^ treatment, attributed to the fact that the exosomes were derived from hepatocytes with functional *Ldlr* mRNA encapsulated (Figure 2D).

Besides the liver, *Ldlr* mRNA was also found in other organs, such as the lung, spleen, kidney, and heart (Figure S5). In contrast, *Ldlr* protein expression was only detected in the liver, lung, kidney, and spleen (Figure 4E-F and S6A-H). The apparent discrepancy could be explained by the detection sensitivity. These data indicated that Exo^Ldlr^ could deliver *Ldlr* mRNA into the liver and some other organs, where the mRNA could be translated to protein.

### Exo^Ldlr^ treatment reduces liver steatosis and atherosclerosis in *Ldlr*^-/-^ mice

**Figure 5:**
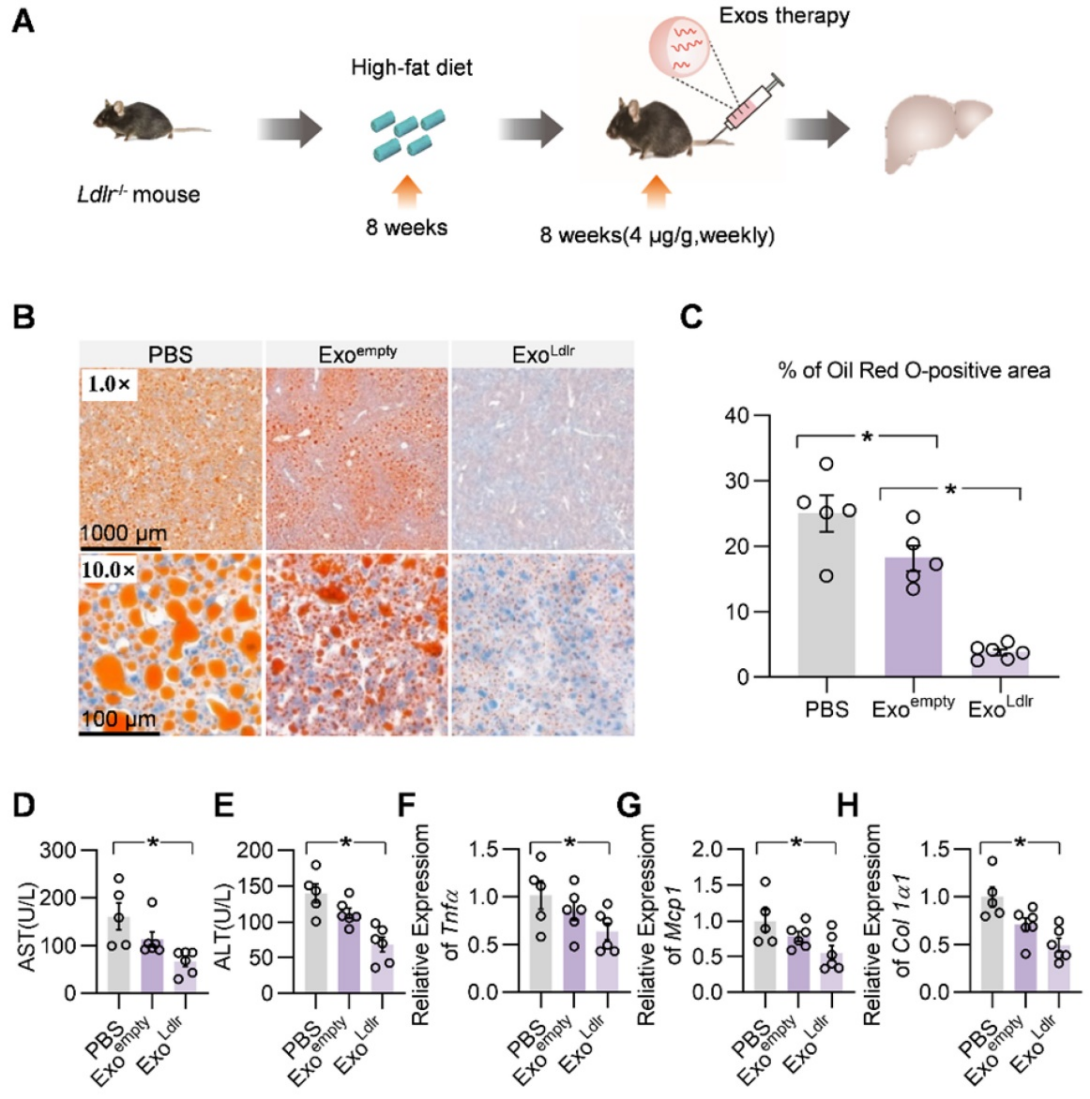
** Exo^Ldlr^ effectively reduces liver steatosis in *Ldlr*^-/-^ mice.** A. Schematic illustration of the experimental procedure. *Ldlr*^-/-^ mice were fed with a high-fat diet for 8 weeks, followed by the injection of indicated exosomes once a week for 8 weeks. At the end of the experiment, lipid deposition in the liver and liver function were examined. B. Representative images of Oil Red O staining of the liver samples from indicated groups. C. Percentage of Oil Red O positive area in livers from indicated groups. (D-E) Plasma ALT (D) and AST (E) levels in *Ldlr*^-/-^ mice treated as indicated. (F-H) qPCR analysis of the expression of *Tnfα* (F), *Mcp1* (G), and *Col1a1* (H) in *Ldlr*^-/-^ mice treated as indicated. Data are expressed as mean ± SEM. *, p < 0.05 by one-way ANOVA. ALT, Alanine aminotransferase; AST, Aspartate aminotransferase.

We systemically analyzed the therapeutic effects of Exo^Ldlr^. The *Ldlr*^-/-^ mice were fed with a high-fat diet for 8 weeks, followed by an injection of indicated exosomes once a week for 8 weeks (Figure 5A). Oil Red O staining of liver sections showed that Exo^Ldlr^ treatment significantly reduced the accumulation of lipid droplets in the hepatocytes (Figure 5B-C). Plasma AST and ALT activities were much lower in Exo^Ldlr^ treated mice (Figure 5D-E). Moreover, the qPCR analysis revealed that Exo^Ldlr^ treatment significantly reduced the expression of fibrogenic and inflammatory genes (Figure 5F-H). HE staining of the liver section and other organs further confirmed that exosome treatment did not cause any noticeable toxic effects in these organs (Figure S7).

**Figure 6:**
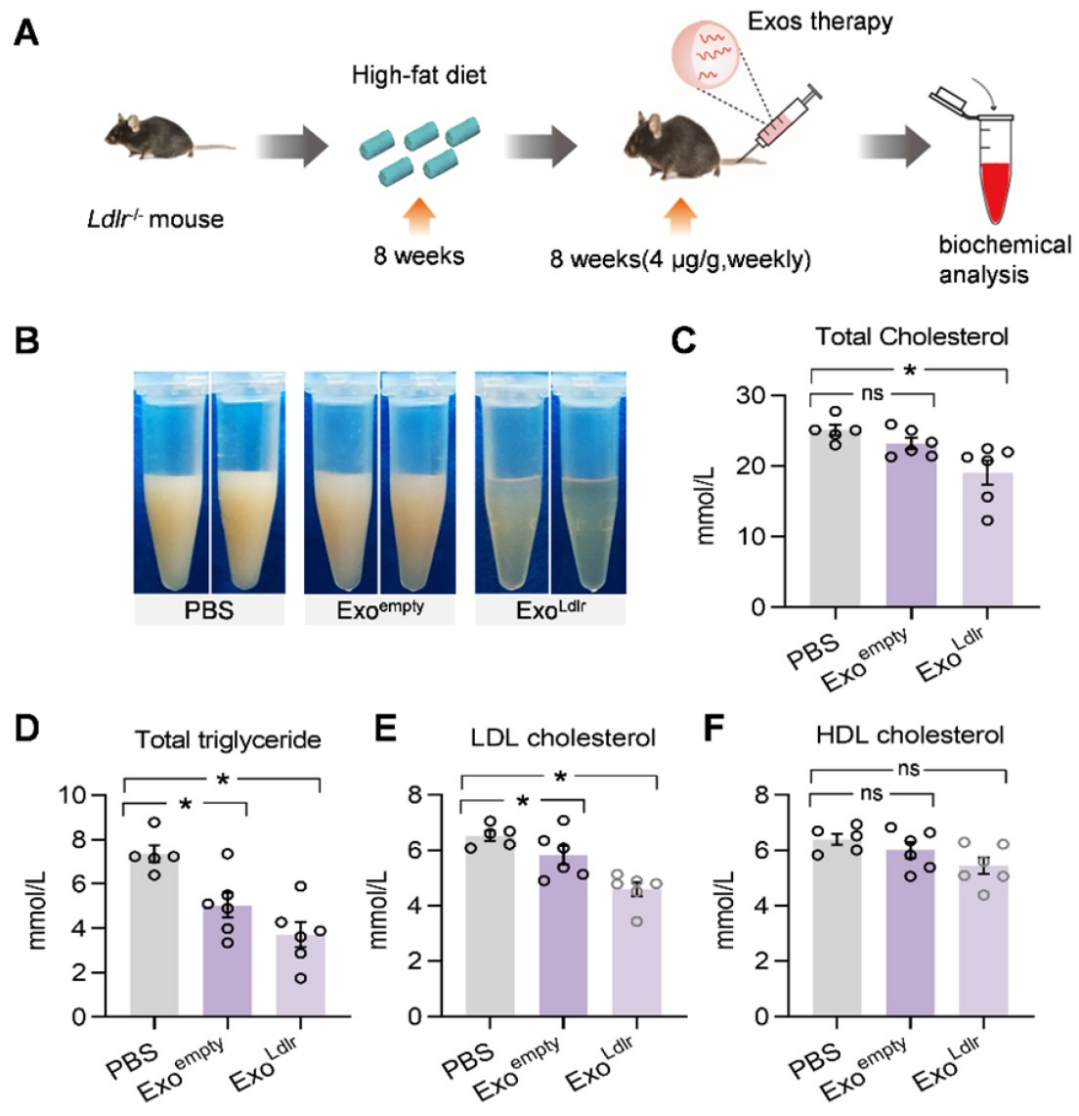
** Exo^Ldlr^ reduces the LDL level in *Ldlr*^-/-^ mice.** A. Schematic illustration of the experimental procedure. *Ldlr*^-/-^ mice were fed with high fat-diet for 8 weeks, followed by injection of indicated exosomes once a week for 8 weeks. B. Representative images of the appearance of serum samples from *Ldlr*^-/-^ mice treated as indicated. (C-F) Examination of the total cholesterol (C), total triglyceride (D), LDL-C (E), and HDL-C (F) in *Ldlr*^-/-^ mice treated as indicated. Data are expressed as mean ± SEM. *, p < 0.05 by one-way ANOVA. ns, no significant difference.

Consistent with the phenotype change in the liver, a striking difference in the appearance of serum was seen after Exo^Ldlr^ treatment. In the control mice receiving PBS treatment, the sera were milky and white. The milky sera were slightly changed after Exo^empty^ treatment. In contrast, the sera were nearly transparent in Exo^Ldlr^-treated mice (Figure 6B). Similarly, Exo^Ldlr^ treatment dramatically decreased total cholesterol, triglyceride, and LDL-C levels (Figure 6C-E). In contrast, no significant change in HDL-C was observed after Exo^Ldlr^ treatment (Figure 6F). Exo^empty^ treatment also reduced the LDL-C level, though to a much smaller extent (Figure 6E). The LDL-C and HDL-C were examined using chemistry analyzer Chemray-800 and not by FPLC that provides more accurate detection of the lipid distribution.

**Figure 7:**
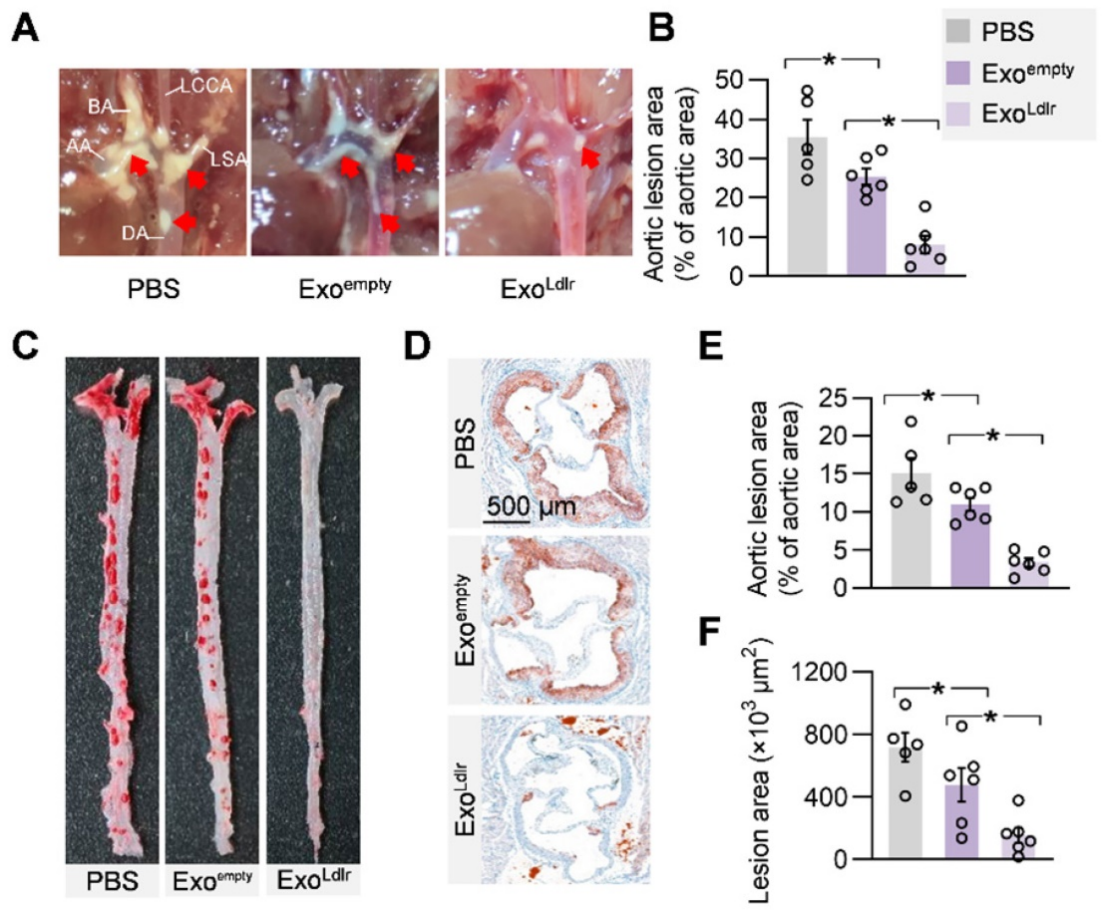
** Exo^Ldlr^ alleviates atherosclerotic lesions in *Ldlr*^-/-^ mice.** A. Representative aortic arch view of the atherosclerotic lesions in *Ldlr*^-/-^ mice treated as indicated. AA, ascending aorta; BA, brachiocephalic artery; LCCA, left common carotid artery; LSA, left subclavian artery; DA, descending aorta. B. Percentage of the atherosclerotic area in the aortic arch. Data are expressed as mean ± SEM. *, p < 0.05 by one-way ANOVA. C. Representative images of Oil Red O staining of the atherogenic lesion areas in mice treated as above. D. Representative images of the cross-sectional view of the aortic roots stained with Oil red O from *Ldlr*^-/-^ mice treated as indicated. E. Percentage analysis of the atherosclerotic region from C. F. Statistical data of the oil O red positive plaque area from D. Data are expressed as mean ± SEM. *, p < 0.05 by one-way ANOVA.

Given the causal role of non-HDL cholesterol in atherosclerosis, we next examined whether Exo^Ldlr^ treatment had any effect on the progression of atherosclerosis lesions. Treatment of control or Exo^Ldlr^ had no noticeable toxic effects on the liver, lung, kidney, heart, and spleen, as determined by HE staining (Figure S7). Notably, the fatty liver was significantly alleviated after the Exo^Ldlr^ treatment (Figure S7). Besides, fewer and smaller atherosclerosis plaques could be observed in Exo^Ldlr^-treated mice (Figure 7A-B and S8A). Furthermore, Oil Red O staining of both aortic tree and aortas root showed that the atherosclerotic plaque burden, especially the lipid core, was much less in the Exo^Ldlr^ group than in the control group (Figure 7C-E), which was further confirmed by the cross-sectional view of the aortic roots (Figure 7D-F).

Besides the reduced lipid core, inflammation and collagen content were also examined in the plaques after Exo^Ldlr^ treatment by CD68 immunostaining and Masson's trichrome staining (Figure S8). There were abundant CD68+ cells in the plaques of the control mice, while the macrophage infiltration was dramatically decreased by the Exo^Ldlr^ treatment (Figure S8B and S8D). Notably, no differences were observed in the collagen content between Exo^Ldlr^-treated and control mice (Figure S8C and S8E).

**Figure 8:**
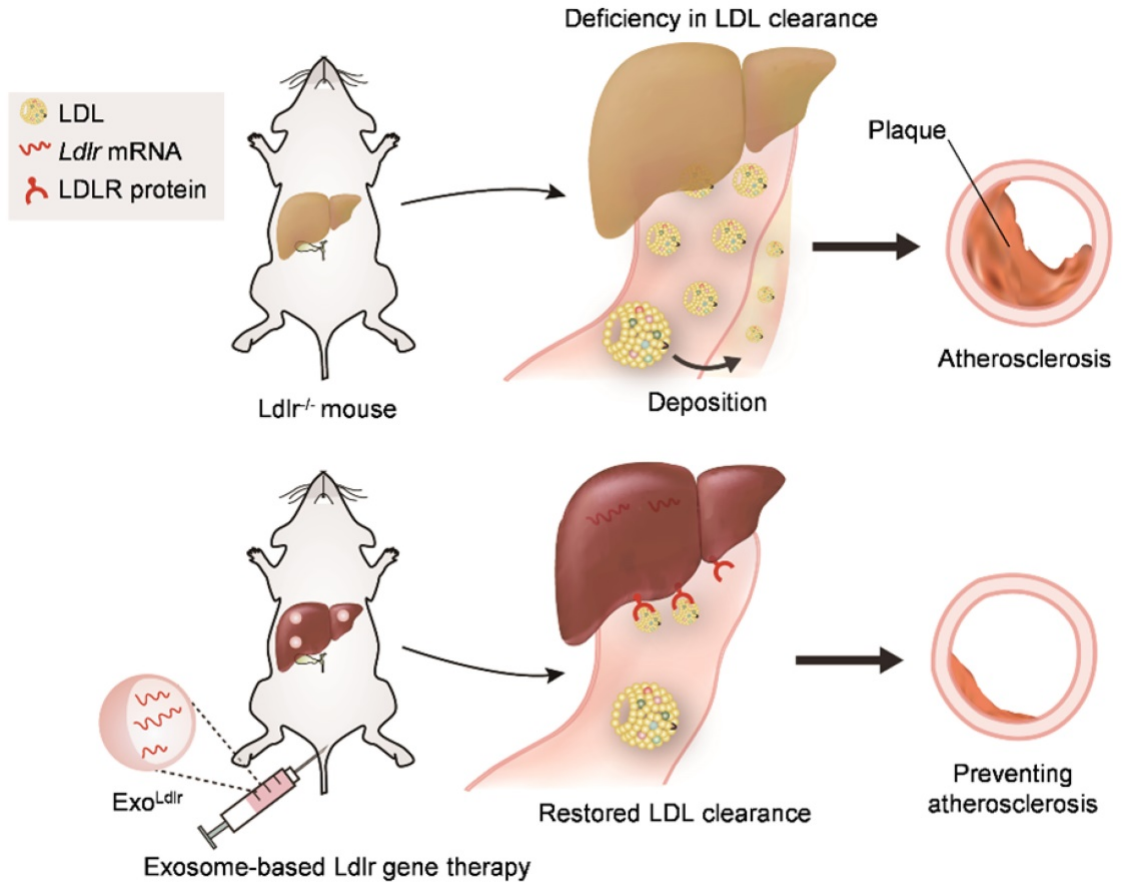
** Schematic illustration of the study.** In *Ldlr*^-/-^ mice, deficiency of *Ldlr* results in abnormal lipid metabolism and atherosclerosis. However, exosome-mediated *Ldlr* mRNA delivery could robustly restore *Ldlr* expression and thus reverse the phenotype.

In summary, we found that LDLR deficiency in *Ldlr*^-/-^ mice resulted in abnormal lipid metabolism and atherosclerosis. However, exosome-mediated *Ldlr* mRNA delivery could robustly restore the expression of LDLR and thus reverse the phenotype, such as steatosis, high LDL-C, and atherosclerosis (Figure 8). Our study has provided a new therapeutic approach for the treatment of FH patients. Significantly, it also shed light on the management of atherosclerosis and other genetic diseases associated with liver abnormity.

## Discussion

In the present study, we engineered, for the first time, an exosomal prodrug by encapsulating the therapeutic *Ldlr* mRNA. We found that exosome-mediated *Ldlr* mRNA delivery could robustly restore *Ldlr* expression in the *Ldlr*^-/-^ mose model, reversing the phenotype of steatosis, high LDL cholesterol, and atherosclerosis.

HoFH remains a medical challenge. LDL-apheresis, similar to kidney dialysis, is currently the last option for patient survival 21. However, the therapy is weekly needed at a high cost. Despite the availability of mechanistic information on the disease, there are no effective drugs for HoFH. LDLR is a membrane-bound protein abundantly expressed on liver cells, removing most of the excess cholesterol from the serum 4. The number of LDLR in the liver determines how quickly cholesterol is removed from the bloodstream. There are no *in vivo* substitutes for LDLR, the key executor of LDL clearance 5, 6. Since it is a membrane protein, therapeutic delivery of LDLR is not feasible, though delivery of the gene or correcting the mutation hold promise. A recent study using CRISPR/Cas9 technology to correct the mutation 22 represents a new gene therapy avenue to cure HoFH. However, the gene-editing efficiency of Cas9 is relatively low 23,24, and the functionality of corrected cells remains a great concern. Furthermore, the delivery of the CRISPR/Cas9 system into the targeted cells is challenging. Unlike the one-time gene correction strategy, we explored the repeated exosomal delivery as an alternative method, which would be advantageous in clinical translation.

Liposomes and viruses are commonly used as carriers of gene therapy, although both have some inherent limitations. For example, viral vectors cause a significant immune response and carry a high risk of cancer if integrated into the genome, whereas the macrophages rapidly clear liposomes as foreign nanoparticles. In contrast, the exosome-based mRNA delivery strategy we proposed has multiple advantages and might be suitable for repeated delivery. First, exosomes can be engineered in patient-specific cells without an immune response, making repeated therapy possible 25. Second, the encapsulated cargo we chose was mRNA, rather than DNA or protein. The mRNA cargo as a therapeutic gene drug has many benefits, such as safety as opposed to the toxicity of cytoplasmic DNA 26. Also, mRNA could be repeatedly translated into multiple protein molecules. Third, *Ldlr* is a rational target for exosome-based RNA delivery. Native *Ldlr* mRNA is unstable due to the stretch of AU-rich elements in the 3'UTR 27. Therefore, replacing the native 3'UTR with the 3'UTR in the vector would increase the mRNA stability, making the delivered LDLR more potent than the endogenous one. Importantly, LDLR is mainly expressed in the liver, the organ dominantly uptaking native exosomes 28,29. Despite multiple advantages, the outstanding challenge is of improving the yield of therapeutic.

Besides hepatocytes, *Ldlr* mRNA is also delivered into the immune cells, although at a much lower level. The expression of *Ldlr* has been reported in immune cells and in other organs, including lung, kidney, heart, and spleen 30-33, where it functions in LDL uptake. The distribution of exosome-mediated *Ldlr* delivery is consistent with the *Ldlr* expression profile in vivo. As the *Ldl-*/- mice are *Ldlr* deficient in the entire body, the *Ldlr* delivery in organs besides the liver, such as lung, kidney, heart, and spleen, would be protective.

Before the clinical application of exosome-mediated *Ldlr* delivery, several concerns need to be addressed.

1) *Ldlr* is widely expressed in many cells and tissues. Improving the delivery efficiency to these cells might be achieved by surface protein engineering. For example, αv integrin-specific RGD showed better targeting and therapeutic response in mammary tumor 34 and RVG-modified exosomes efficiently targeted ischemic areas in the brain 35.

2) Reducing off-target delivery is important. It has been reported that some exosomes are engulfed by macrophages rather than parenchymal cells in the liver 36. The off-target delivery not only causes side effects, but also leads to an increase in the dosage of exosomes required for treatment. Blocking the off-target endocytosis of therapeutic exosomes is, therefore, desirable.

3) Although *Ldlr* is stable, developing *Ldlr* mRNA with longer stability is still needed.

4) It would be beneficial to encapsulate the mRNA into exosomes in a cell-free system. Electroporation is used to load small RNAs, such as siRNA 37,38 and miRNA 39, while the mRNA or lncRNA loading efficiency remains poor. Also, exosomes can be harnessed from serum for drug loading.

5) Improving the yield of exosomes at a high purity is urgently needed. In this context, booster and microchip strategies have recently been used 17,40.

Most of these concerns represent common obstacles for gene therapy. Exosome-based gene therapy is currently in its phase I clinical trial (ClinicalTrials.gov identifier: NCT03608631) to treat pancreatic cancer associated with Kras G12D mutation. Therefore, therapeutic trials of Exo^Ldlr^ in FH and other associated genetic diseases can be envisioned in the future.

Our study also provides useful information for the management of atherosclerosis and genetic liver diseases. The main cause of the mortal myocardial infarction and stroke is the rupture of vulnerable atherosclerotic plaques 41,42. Our results showed that Exo^Ldlr^ treatment minimized and stabilized atherosclerotic plaques, for which the underlying mechanisms remain to be elucidated. Consistent with our findings, it has been shown that long-term lipid-lowering reduced the lesion size and also contributed to the stability of atherosclerotic plaques 43. Thus, exosome-mediated *Ldlr* mRNA delivery might be a promising strategy for refractory LDL-C and vulnerable plaques. There is a wide spectrum of genetic liver diseases worldwide, such as ornithine transcarbamylase deficiency and Wilson's disease. Despite the low incidence of these diseases, a considerable overall affected population exists 44,45. Since the liver is the targeted organ, the exosome strategy proposed here could provide an alternative therapy for these inherited disorders.

Taken together, our study provided evidence that exosome-mediated *Ldlr* mRNA delivery could robustly restore LDLR expression and reverse the phenotypes, such as steatosis, high LDL-C, and atherosclerosis (Figure 8). The study represents a new therapeutic approach for the treatment of FH patients and might also be useful for managing atherosclerosis and other genetic diseases associated with liver abnormity.

## Materials and Methods

### Cell culture and treatments

HEK293T cells and mouse liver 12 (AML12) cells were grown in DMEM high glucose medium (Logan, Utah, U.S.A.), supplemented with 10% fetal bovine serum (FBS) and 1% antibiotics (Logan, Utah, U.S.A.). Fresh medium was added to the cells every 2 days and maintained at 37 °C in 5% CO2.

### Plasmid Construction

Total RNA was extracted from mouse liver tissues and reverse transcribed to cDNA by Transcriptor Reverse Transcriptase (Indianapolis, IN, U.S.A.). The CDS of the *Ldlr* cDNA was amplified by specific primers flanked with Pac I (forward) and BstB I (reverse). The amplicon was digested and cloned into the pWPI vector. The right clones were confirmed by DNA sequencing and stored for the following application. PCR primers used are listed in Table S1.

### Cell transfection, infection, and exosome isolation

AML12 cells were plated in 6-well plates one day before transfection or infection. For transfection, cells were transfected with 4 μg control or *Ldlr*-expressing vectors by Lipofectamine 2000 (Invitrogen) according to the manufacturer's instructions. For virus package, HEK293T cells at 60%-80% confluency were transfected with control or *Ldlr*-expressing vectors, together with the standard packaging plasmid (psPAX2), or envelope plasmid (pMD2.G) at a 4:3:1 mass ratio by Lipofectamine 2000. Cells were then incubated at 37 ℃, 5% CO_2_ and the lentiviruses in supernatants were collected after 72 h. For virus infection, AML12 cells were infected with the lentiviruses in the medium containing 8 μg/mL polybrene (Sigma, St. Louis, USA), at MOI of 50. Twelve hours later, cells were maintained in the DMEM medium with 10% exosome-free FBS for another 24-36 h, followed by exosome isolation.

The exosomes were isolated as described previously, with some minor modifications 46. Exoquick^TC^ kit was chosen for exosome isolation in this study, though some proteins and nucleic acid/protein complexes are possibly coprecipitated in this method. Our preliminary data revealed that the remnant nucleic acid/protein complexes had minimal effects on the loading efficiency and in vivo function. Briefly, supernatants were centrifuged at 500 g for 10 min and then 10000 g for 20 min to remove cells and debris, respectively. The resulting supernatant was then filtered through 0.4 μm filters, followed by exosomes harvest using Exoquick^TC^ kit as per manufacturer's instructions. Isolated exosomes were resuspended in PBS or DMEM and stored at -80 °C till use. To avoid exosome differences among batches, we pooled the exosomes from different batches before in vivo experiments.

### Exosome Characterization

Purified exosomes from AML12 cells were resuspended in PBS and then dropped onto the metal grid. After staining with 2% uranyl acetate, the exosomes were dried for 0.5 h. The exosomes were then examined using the JEM-2000EX electron microscope (Tokyo, Japan) and images were taken by an armed camera.

For nanoparticle tracking analysis, purified exosomes from different sources were diluted to 5 ng/μl, followed by analysis on the Particle Metrix instrument.

### LDL clearance assay

AML12 cells in 6-well plates were incubated with 40 μg indicated exosomes for 24 h. Subsequently, the cells were washed with PBS and cultured in DMEM without serum containing 5 μg/mL DiI-LDL (770230-9, Kalen Biomedical) at 37 ℃. DiI-LDL uptake was analyzed by a fluorescence microscope, and the nuclei were counterstained with Hoechst.

### Animal experiments

All animal experiments were performed under protocols approved by the Animal Care and Use Committee of Fourth Military Medical University. *Ldlr*^-/-^ mice (C57/BL background) were purchased from the Model Animal Research Center of Nanjing University and fed a high-fat diet for 8 weeks before the experiments.

For *in vivo* analysis of exosomes distribution, purified exosomes were incubated with DiR at the final concentration of 8 μM (Invitrogen) for 30 min at 37 °C. Free DiR was removed by another round of exosome isolation. DiR-labeled exosomes (DiR-Exos) were injected through the tail vein, and the distribution of exosomes was analyzed by in vivo imaging system (IVIS) 4 h after injection.

For analysis of the intracellular distribution of exosomes, DiI-labeled exosomes were prepared similarly before tail vein injection. Four hours after injection, mice were sacrificed and isolated tissues were fixed for 15 min by 4% paraformaldehyde before sectioning. Nuclei were counterstained with Hoechst (Invitrogen), and the DiI-labeled exosomes were tracked under a fluorescence microscope (ECLIPSE Ti, Nikon, Tokyo, Japan). The entire process was conducted in the dark.

For analysis of delivery efficiency, exosomes at a dose of 4 μg/g were injected via the tail vein. Three days post-injection, mice were sacrificed and the main organs (heart, liver, spleen, lung, and kidney) were isolated and subjected to qPCR and Western blot analysis of *Ldlr* abundance.

In therapeutic intervention using exosomes, 8-week old *Ldlr*^-/-^ mice were fed a high-fat diet for 8 weeks before injection with PBS, control, or therapeutic exosomes at a dose of 4 μg/g weekly for 8 weeks. At the end of the experiments, mice were euthanized after 8 h. Blood, liver and aortae were isolated and processed for further analysis.

### Western blotting

Samples were prepared with RIPA Lysis Buffer (Beijing, China) at 4 °C for 30 min. The protein concentration in each sample was determined by the Pierce BCA Protein Assay Kit (Thermo, U.S.A.). Equal amounts of proteins were then concentrated on SDS-PAGE (6%) and separated by SDS-PAGE (12%), followed by transfer onto nitrocellulose filter membranes. After blocking with 3% BSA, membranes were incubated with primary antibodies, anti-LDLR (ab52818, Abcam), anti-GM130 (sc71166, Santa Cruz), anti-TSG101 (ab83, Abcam), anti-CD9 (ab92726, Abcam), or anti-GAPDH (D110016-0100, BBI life sciences) overnight at 4 °C. After washing three times in TBST, the membranes were incubated with anti-rabbit (7074, CST) or anti-mouse (7076, CST) secondary antibodies corresponding to the primary antibodies at room temperature for 1h.

### Polymerase Chain Reaction

Total RNA was extracted using TRIzol reagent (Invitrogen, IN, U.S.A.). Reverse- transcription was performed by Transcriptor Reverse Transcriptase (Indianapolis, IN, U.S.A.) according to manufacturers' instructions. Quantitative real-time PCR was performed by the FastStart Essential DNA Green Master (Indianapolis, IN, U.S.A.). Relative gene expression was normalized to GAPDH and quantified with the 2^ΔΔCt^ method for comparison. Semi-quantitative PCR analysis was run similarly, the amplicons were separated on an agarose gel, and the sizes of the bands were detected under UV.

To differentiate between the wild-type *Ldlr* mRNA and the endogenous mutant *Ldlr*, a nested real-time PCR assay was developed to increase the sensitivity and specificity. The external primer set spanned the region of the deleted region, while the internal primer set was localized within the deleted region. cDNA samples were first run with the external primers (0.1 pM) for 5-10 cycles and then 1/20 of the products were used as the template for PCR with internal primers. The sequences of PCR primers were provided in Table S1.

### Serum biochemistry

Blood samples were collected by extracting the eyeball after 8 h of fasting. The concentration of ALT (Alanine Aminotransferase), AST (Aspartate Aminotransferase), plasma triglycerides, total cholesterol, HDL cholesterol, and LDL cholesterol levels were measured by Chemray 800 at Wuhan Servicebio technology CO., LTD.

### Histology

The mice were anesthetized with 120 mg/kg body weight of ketamine and 24 mg/kg body weight xylazine in a vehicle containing 0.9% sodium chloride. The mice were perfused with PBS and thereafter the organs, including the liver, lung, spleen, kidney, and heart, were dissected. The dissected organs were post-fixed at 4% paraformaldehyde for 1 h.

Liver sections were prepared for H&E and Oil-red-O staining. The heart and aorta were exposed and cleaned from surrounding fat tissues. Aortic arch bifurcation images were captured by a digital camera equipped on the stereomicroscope. Subsequently, the aorta-to-iliac bifurcation was isolated and split along the midline and stained with Oil-red-O. The percentage of lesion area was calculated as the total lesion area divided by total surface area using Image J. For the atherosclerotic lesions within the aortic valve (aortic sinus), the samples were fixed with 4% paraformaldehyde and transferred to PBS with 30% sucrose overnight. The samples were embedded in OCT compound and sectioned at 10 μm. The sections were then stained with H&E and Oil-red-O to compare the lesion size and lipid core area among groups.

For immunohistochemical staining of CD68+ macrophages, sections were first blocked with 4% bovine serum albumin. The sections were then incubated with anti-CD68 antibody overnight (4 °C in a humidified chamber) and washed, followed by incubation with HRP-conjugated secondary antibody for 1 hour at 37 °C. Subsequently, the sections were developed with DAB and visualized under a microscope. Images were captured and the CD68-positive area was quantified by ImageJ.

### Statistical Analysis

Data were expressed as mean ± SEM. Shapiro-Wilk test was used to determine data distribution normality. Student t-test or the Mann-Whitney U test were used for two-group comparison for normally distributed data or abnormally distributed data, respectively. For multiple comparisons among three or more groups, one- way ANOVA was conducted followed by Tukey's posthoc test (Graphpad Prism 7.0). P values of <0.05 indicate statistical significance.

## Supplementary Material

Supplementary figures and table.
