# Supplementary material for: Exosome-based Ldlr gene therapy for familial hypercholesterolemia in a mouse model
Source: Theranostics. 2021 Jan 1;11(6):2953–65. doi: 10.7150/thno.49874 (PMC7806494; doi:10.7150/thno.49874)
Supplement: Supplementary file 1 — Supplementary figures and table. [file thnov11p2953s1.pdf]

Supplementary Information

Exosome-based *Ldlr*-gene therapy for familial hypercholesterolemia in a mouse model

Zhelong Li[1,2,#], Ping Zhao[1,#], Yajun Zhang[3,#], Jia Wang[1], Chen Wang[1,2], Yunnan Liu[1,2], Guodong Yang[2,*], Lijun Yuan[1,*]

[1]Department of Ultrasound Diagnostics, Tangdu Hospital, Fourth Military Medical University, Xi'an, People's Republic of China

[2]The State Laboratory of Cancer Biology, Department of Biochemistry and Molecular Biology, Fourth Military Medical University, Xi'an, People's Republic of China

[3]Department of Ultrasound Diagnostics, Beijing Tongren Hospital, Capital Medical University, Beijing 100730, China

# Equal Contribution.

* Correspondence authors:

Lijun Yuan, Department of Ultrasound Diagnostics, Tangdu Hospital, Fourth Military Medical University, Xinshi Road NO.569th, 710038, Xi'an, China, email: yuanlj@fmmu.edu.cn; Tel: +862984777471, Fax: +862984777471.

Guodong Yang, The State Laboratory of Cancer Biology, Department of Biochemistry and Molecular Biology, Fourth Military Medical University, Changlexi Road NO.169th, 710032, Xi'an, email: yanggd@fmmu.edu.cn; Tel: +862984774516, Fax: +862984774516.

Table S1: Sequences of PCR primers

Name		Sequence
qPCR		
Ldlr	Forward	5’- TGACTCAGACGAACAAGGCTG-3’
	Reverse	5’- CTAACTAAACACCAGACAGAGGC-3’
Mouse *Gapdh*	Forward	5’-AGGTCGGTGTGAACGGATTTG-3’
	Reverse	5’-TGTAGACCATGTAGTTGAGGTCA-3’
Col1a1	Forward	5'-GCTCCTCTTAGGGGCCACT-3'
	Reverse	5'-ATTGGGGACCCTTAGGCCAT-3'
Tnfα	Forward	5’-GACGTGGAACTGGCAGAAGAG-3’
	Reverse	5’-TTGGTGGTTTGTGAGTGTGAG-3’
Mcp1	Forward	5’-CCTGCTGTTCACAGTTGCC-3’
	Reverse	5’-ATTGGGATCATCTTGCTGGT-3’
Human GAPDH	Forward	5’-GGAGCGAGATCCCTCCAAAAT-3’
	Reverse	5’-GGCTGTTGTCATACTTCTCATGG-3’
**Nested PCR**		
External primer of *Ldlr*	Forward	5’- CTCCCAGGATGACTTCCGAT -3’
	Reverse	5’- CGCAGTGCTCCTCATCTGAC -3’
Internal primer of *Ldlr*	Forward	5'- CGACGGGGATGTCGACTGTGTTGA -3'
	Reverse	5'- TCGGCCCTGGCAGTTCTGTG -3'
Cloning		
Ldlr	Forward	5’-GGTTAATTAACAATGAGCACCGCGGATCTGA-3’
	Reverse	5’-GGTTCGAATCATGCCACATCGTCCTCCAGGC-3’

Supplementary Figure Legends

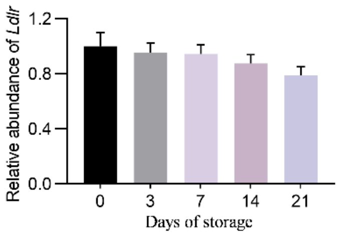

Figure S1: Stability of the *Ldlr* mRNA in the exosomes. qPCR analysis of the *Ldlr* mRNA in the exosomes. About 10^9 exosomes were preserved for indicated periods, followed by RNA isolation. Relative abundance was analyzed by qPCR and normalized to the day 0 sample.

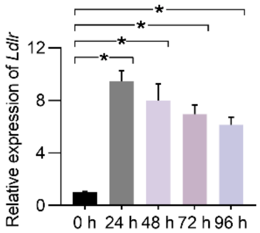

Figure S2: Functional duration of the delivered *Ldlr* mRNA in recipient cells. qPCR analysis of the *Ldlr* mRNA in recipient cells following incubation with exosomes. About 10^6 cells were incubated with 40 μg exosomes and harvested at different times post-incubation. Data are expressed as mean ± SEM. *, $p < 0.05$ by one-way ANOVA.

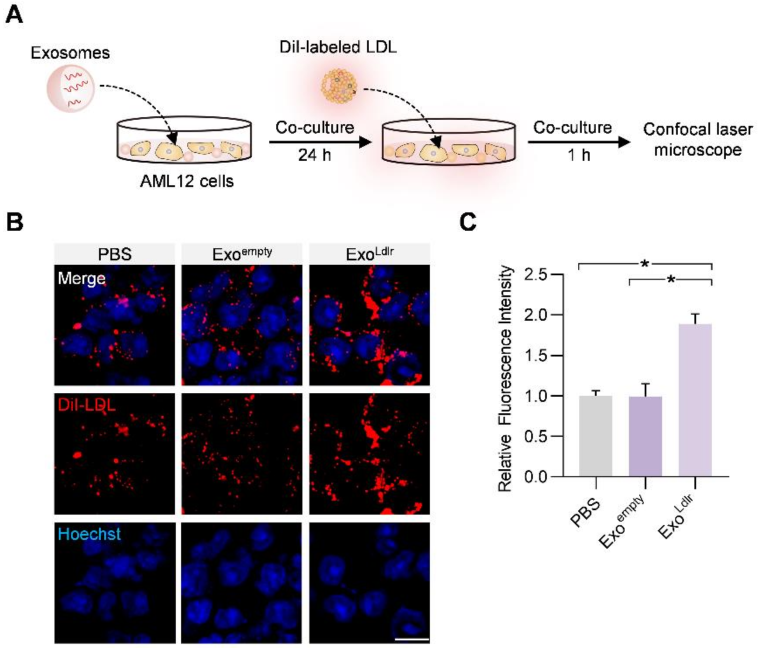

Figure S3: Exo^{Ldlr} promote LDL uptake in AML12 cells. A. Schematic representation of the

experimental procedure. AML12 cells were treated with indicated exosomes followed by incubation with 5 μg/ml DiI-labeled LDL 24 h later. LDL uptake was visualized by confocal microscopy at 1 h post-incubation. B. Fluorescence images of DiI-labeled LDL taken up by cells with different treatments. Nuclei were counterstained with Hoechst. PBS served as negative control. Scale bar=10 μm. C. Quantification of the fluorescence signal intensity. Data are expressed as mean ± SEM of three independent experiments. At least 100 cells were counted in each group per replicate. *, $p < 0.05$ by one-way ANOVA.

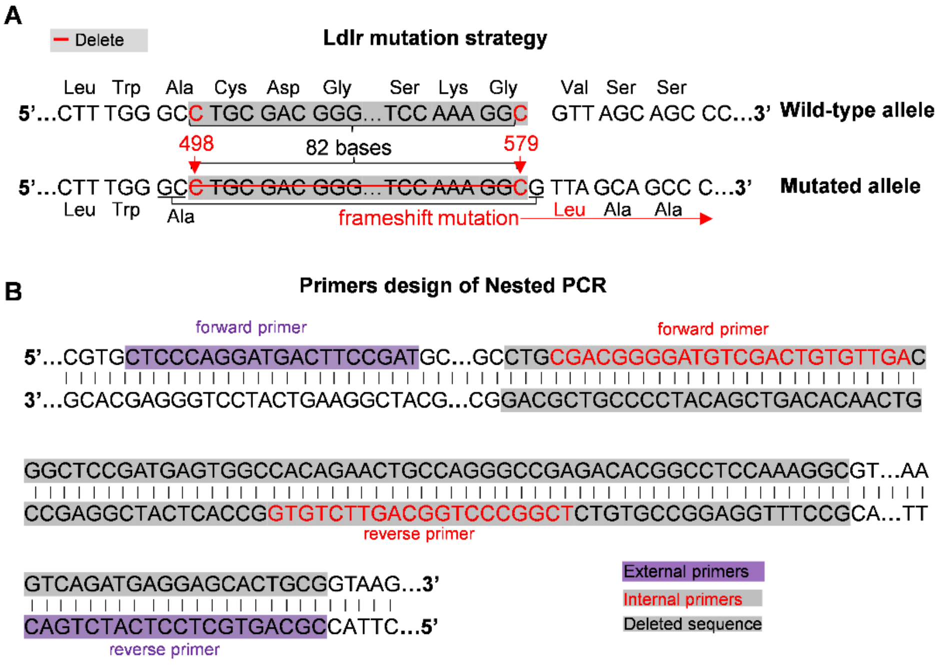

Figure S4: Illustration of *Ldlr* gene deletion strategy and primer design. A. *Ldlr* gene deletion strategy. In the knockout mice, an 82 bp sequence was deleted, resulting in a frameshift. B. Schematic representation of the location of the primers for nested PCR and qPCR.

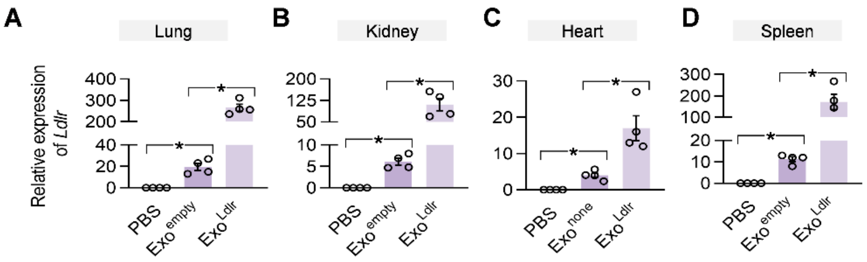

Figure S5: RNA expression of *Ldlr* in different organs of mice receiving exosome therapy. (A-D) qPCR analysis of wildtype of *Ldlr* mRNA in the lung (A), kidney (B), heart (C), and spleen (D). *Ldlr*$^{-/-}$ mice were fed with a high-fat diet for 8 weeks, followed by injection of indicated exosomes. *Ldlr* expression at mRNA and protein levels was examined 3 days after injection. Data are

expressed as mean ± SEM. *, $p < 0.05$ by one-way ANOVA.

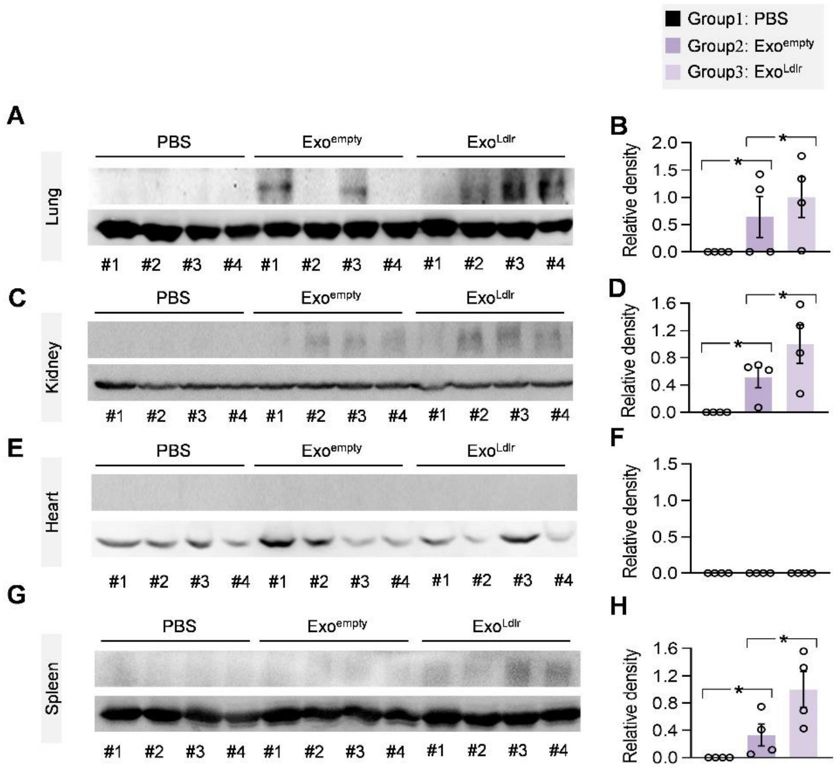

Figure S6: Protein expression of LDLR in various organs of mice receiving exosome therapy. (A-H) Western blot analysis of the LDLR expression in the lung (A), kidney (C), heart (E), and spleen (G) from mice treated as indicated. Quantification analysis of the Western blot bands by densitometry in B, D, F, and H. Data are expressed as mean ± SEM. *, $p < 0.05$ by one-way ANOVA.

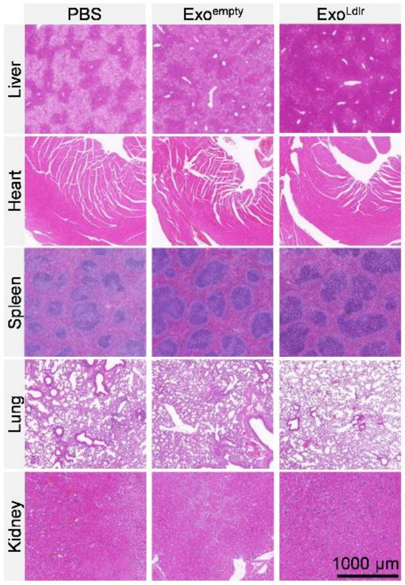

Figure S7 H&E staining of different organs from mice with indicated groups. No significant

toxicity was observed in the liver, heart, spleen, lung, and kidney from the HE staining results.

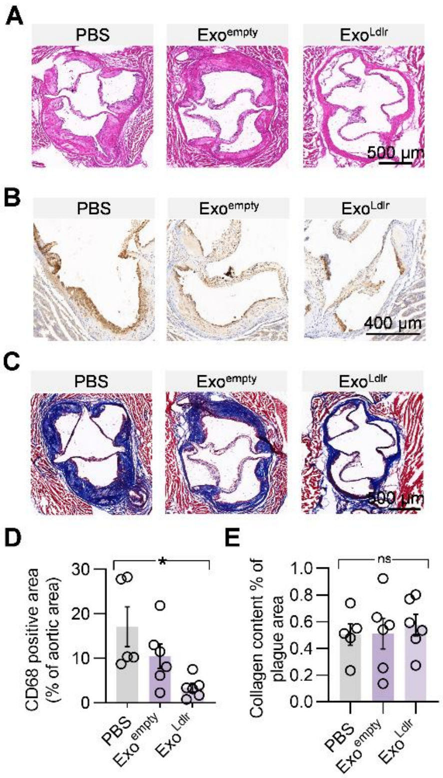

Figure S8: Exo^{Ldlr} reduces the inflammation of atherosclerotic plaques. A. H&E staining of aortic root sections from mice treated as indicated. Scale bar=500 μm. B. CD68 immunostaining in aortic root sections from mice treated as indicated. Scale bar=400 μm. C. Representative Masson's trichrome staining images of an aortic sinus. Scale bar=500 μm. D. Percentage of CD68-positive area in the plaque. Data are expressed as mean ± SEM. *, $p < 0.05$ by one-way ANOVA. E. Percentage of collagen content in the plaque. Data are expressed as mean ± SEM. ns, no significance.
